# Radiotherapeutic management of cervical lymph node metastases from an unknown primary site – experiences from a large cohort treated with modern radiation techniques

**DOI:** 10.1186/s13014-020-01529-z

**Published:** 2020-04-15

**Authors:** Tanja Sprave, Alexander Rühle, Katharina Hees, Tobias Kalckreuth, Vivek Verma, Raluca Stoian, Constantinos Zamboglou, Jens Pfeiffer, Roland Laszig, Andreas Knopf, Anca-Ligia Grosu, Nils H. Nicolay

**Affiliations:** 1 Department of Radiation Oncology, Medical Center, Faculty of Medicine, University of Freiburg, Robert-Koch-Str. 3, 79106 Freiburg, Germany; 2 German Cancer Consortium (DKTK) Partner Site Freiburg, German Cancer Research Center (dkfz), Heidelberg, Germany; 3 Department of Statistics, TU Dortmund University, Dortmund, Germany; 4 Department of Radiation Oncology, Allegheny General Hospital, Pittsburgh, PA USA; 5 Department of Otorhinolaryngology, Medical Center, Faculty of Medicine, University of Freiburg, Freiburg, Germany

**Keywords:** Head-and-neck cancer, Carcinoma of unknown primary, Lymph node, CUP, Radiotherapy, Chemotherapy

## Abstract

**Purpose:**

To analyze management and outcomes following (chemo)radiation therapy in patients with cervical lymph node metastases from an unknown primary site (CCUP) in a large single-center cohort.

**Methods:**

Between 2008 and 2019, 58 patients with CCUP were treated with (chemo)radiation therapy at the University of Freiburg Medical Center and were included in this analysis. Overall survival (OS), locoregional progression-free survival (PFS) and distant metastasis-free survival (DMFS) were calculated using the Kaplan-Meier method. The use of diagnostic procedures and their impact on oncological outcomes was analyzed by Cox regression, and treatment-related toxicities were quantified.

**Results:**

Median follow-up was 29.9 months (range 4.6–121.9). Twenty-one patients (36.2%) received definitive RT, 35 (60.3%) underwent adjuvant RT, and 2 (3.4%) were treated for oligometastatic disease. Concurrent chemotherapy was prescribed in 40 patients (69.0%). 89.6% of patients completed the prescribed RT, and 65.0% completed the prescribed simultaneous chemotherapy. Locoregional recurrence was observed in 7 patients (12.1%) and distant metastases in 13 cases (22.4%). OS was 81,1, 64.9% and 56,6% after 1, 3 and 5 years, respectively.

Univariate analysis of age, gender, extracapsular spread, tumor grading, neck dissection, diagnostic utilization of ^18^F-fluorodeoxyglucose positron-emission tomography and concomitant chemotherapy showed no effect on OS (*p* > 0.05 for all), while smoking was significantly associated with decreased survival (*p* < 0.05). There was a trend towards impaired OS for patients with advanced nodal status (pN3) (*p* = 0.07). Three patients (5.2%) experienced grade 3 radiation dermatitis, and 12 (22.4%) developed grade 3 and 1 (1.7%) grade 4 mucositis.

**Conclusions:**

RT of the panpharynx and cervical lymph nodes with concurrent chemotherapy in case of risk factors demonstrated good locoregional control, but the metachronous occurrence of distant metastases limited survival and must be further addressed.

## Background

Cancer of unknown primary is the seventh most common malignant disease in the Western world and constitutes the fourth most common cause for cancer deaths [1]. Although relatively uncommon, management of cervical lymph node metastases from a cancer of unknown primary (CCUP) remains a therapeutic challenge [2]. This condition most often affects older men with nicotine and/or alcohol abuse, and the most common histology remains squamous cell carcinoma (SCC) [3]. Usually, cervical swelling is the first symptom noted by patients, and pain and dysphagia have also been reported to result in the diagnosis of CCUP [4].

In the last decade, national and international recommendations on standardized procedures for CCUP have been updated [5]. Moreover, refinement and further development of diagnostic and therapeutic procedures have improved management and hence resulted in improved outcomes [6–9]. Diagnostic workup includes careful clinical examination, cervical nodal ultrasound and panendoscopy, combined with diagnostic tonsillectomy. Computed tomography (CT), magnetic resonance imaging (MRI), and ^18^F-fluorodeoxyglucose-positron emission tomography-CT (FDG-PET-CT) are also essential to further clarify the extent of the disease and to assess potential primaries. In this context, the advent of FDG-PET-CT has resulted in detection rates of up to 40% of occult primaries not amenable to conventional diagnostic imaging and also helps to further clarify the extent of the disease [10, 11]. In this regard, FDG-PET-CT has been demonstrated as a cost-effective measure in patients with N1 and N2 status [12].

Today, molecular analyses and assessment of human papillomavirus (HPV) and Epstein-Barr virus (EBV) status provide additional diagnostic and prognostic information. As demonstrated for other SCCs of the head-and-neck region, HPV positivity influences the prognosis of CCUP patients, and the presence of ECS in p16-positive tumors does not seem to affect survival [8]. However, de-escalation approaches for these cases are not recommended outside of clinical trials. The real impact of the available diagnostic means on clinical decision-making and therapeutic approaches remains unclear and illustrates broad differences between individual centers.

In early stage disease (cN1/pN1) without additional risk factors, local single-modality treatment (surgery or radiotherapy) constitutes the treatment standard [13], but for advanced disease, the extent of local therapies remain controversial, especially regarding treatment of elective nodal regions. In the case of radiotherapy, several concepts have been proposed that may encompass partial or total mucosal coverage as well as ipsi- or bilateral lymphatics [14–16].

CCUP management remains a multidisciplinary challenge, owing to the lack of prospective randomized studies. The goal of this analysis was to evaluate patterns of management and resulting oncological outcomes of CCUP in a large single-center patient cohort receiving radiotherapy.

## Methods

### Patients

This analysis included all patients with histologically proven CCUP treated with radiotherapy between 2008 and 2019 at University of Freiburg Medical Center, Germany. Demographic and clinical data were retrospectively taken from electronic patient records. At the time of therapy, no primary tumor had been identified in any patient following the diagnostic workup delineated below. In all patients, therapy was based on recommendations of the multidisciplinary tumor board. Due to the time period included in this analysis, HPV and EBV status was not routinely tested. Pathological data were taken from the pathology reports. Tumor classification was determined on the basis of pathological reports and contrast-enhanced imaging and was encoded according to the 7th edition of the UICC TNM classification. “Smokers” referred to a smoking history of at least 10 years. Ethical approval was obtained from Freiburg University Independent Ethics Committee for this analysis (record no. 555/18).

### Diagnostic procedures and surgery

Primary workup included a detailed clinical examination, contrast-enhanced CT of the neck, thorax and abdomen, and ultrasound of the neck. FDG-PET-CT and MRI of the neck or abdomen were performed at clinical discretion based on availability and time period.

Panendoscopy with esophagoscopy was conducted, and, if not yet performed, a diagnostic tonsillectomy was completed. For limited stages (cN1) without neck dissection due to patient comorbidities or patient wishes, fine needle aspiration (FNA) or selective extirpation of affected lymph nodes were performed. Up-front neck dissection (ND) of the affected sides was completed for all other patients.

### Radiation treatment and chemotherapy

**Fig. 1:**
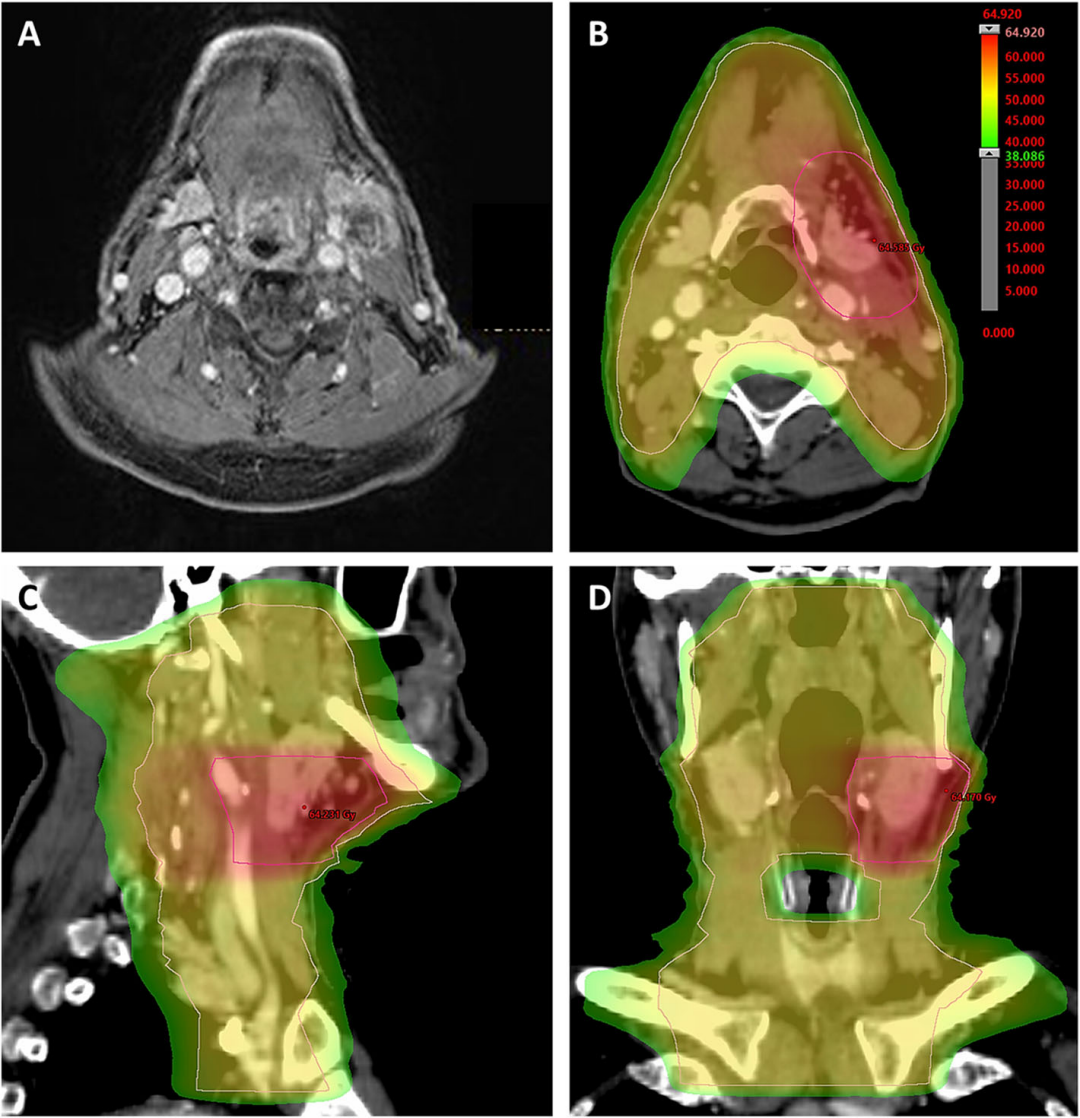
Adjuvant chemoradiotherapy for a CCUP in a 70-year-old male patient. (**A**) Pretherapeutic sonography, CT and MRI imaging (**a**) in December 2017 showed a pathological lymph node in level IIa on the left side and a suspicious lymph node in level II on the right side. A panendoscopy with multiple biopsies of different mucosal regions revealed no primary tumor. As the patient had a tonsillectomy as child, no additional tonsillectomy was performed. The recommended FDG-PET-CT was not conducted, as the costs were not covered by the patient’s health insurance. After bilateral ND in January 2018, pathological assessments showed one necrotic lymph node (1.4 cm diameter) with poorly differentiated (G3), HPV-positive squamous cell carcinoma cells in left-sided level IIa, giving a cTx pN1 cM0 CCUP according to the 7th Edition of the UICC TNM classification. Based on the recommendations of the multidisciplinary tumor board, an adjuvant cisplatin-based chemoradiotherapy with intensity-modulated radiotherapy was performed between March and April 2018. The elective lymphatic drainage and mucosa received 50 Gy in 25 fractions, while the high-risk PTV was treated with a sequential boost of 10 Gy delivered in 5 fractions. (**b, c** and **d**) Dose distribution of a volumetric modulated arc therapy plan in an axial (**b**), sagittal (**c**) and coronary (**d**) scan image. The last follow-up in March 2019 showed no signs of recurrence

Patients received image-guided radiotherapy (IGRT), mainly using intensity-modulated techniques (IMRT) (Fig. 1). All patients were immobilized with a head-neck thermoplastic mask and underwent a planning CT scan. A margin of 0.5–1 cm was added to the gross tumor volume (GTV) for clinical target volume (CTV) delineation in order to treat microscopic spread, and a PTV margin of 0.3–0.5 cm was added for including organ motion and set up-errors. Radiotherapy planning was performed using the Oncentra MasterPlan® (Nucletron BV, Veenendaal, the Netherlands) and Eclipse™ planning systems (Varian Medical Systems). Elective clinical target volumes (CTV) included lymph node levels Ib to V on the affected cervical sides and level II to V for unaffected sides as well as oropharyngeal, hypopharyngeal and laryngeal mucosa. Oral cavity or nasopharyngeal mucosa was included depending on the location of the lymph node metastases.

The elective lymphatic drainage and mucosa was treated to a total dose of 50–54 Gy in five weekly fractions of 1.8–2.0 Gy. Macroscopic lymph nodes (if not resected by ND) received 66–70 Gy using a simultaneous or sequential boost concept. After resection, the surgical lymph node bed was treated to a total dose of 60–66 Gy depending on pathologic findings and the presence of ECS. Risk structures were outlined on the planning CT and protected in accordance with QUANTEC recommendations [17].

### Outcome measures

Follow-up care included clinical examinations including endoscopy and radiological imaging with ultrasound as well as CT and/or MRI. Follow-up examinations including imaging were performed every 3 months for the first 3 years and every 6 months between years 3 and 5. Patients, who were lost to follow-up, were censored for statistical analyses. Local or locoregional relapse, distant progression and overall survival (OS) were analyzed for each patient. OS was defined as the period from the start of therapy to the last contact or death. Based on follow-up radiological imaging, locoregional progression-free survival (PFS) was assessed and defined as the time from treatment initiation to locoregionally progressive disease or death. Distant metastasis-free survival (DMFS) was defined as newly diagnosed distant metastases on follow-up radiological imaging or death.

### Statistical analysis

Statistical analysis was performed with R Studio version 3.6.1. The Kaplan–Meier estimator using log-rank test was applied for OS as well as for PFS and DMFS. Additionally, univariate Cox-regression analyses were performed to assess the effect of several clinical factors on OS (smoking, advanced nodal status, age, gender, ECS, tumor grading, ND and utilization of FDG-PET-CT). A *p*-value of < 0.05 was considered to be statistically significant. Since this was an exploratory trial, all *p*-values were interpreted descriptively and no adjustment for multiple testing was applied.

## Results

### Patient and treatment characteristics

Fifty-eight patients receiving IGRT and mainly IMRT for CCUP were included in this analysis. The median age of the analyzed patient cohort was 62 years (range 37–92), and patients were predominantly male (*n* = 44, 75.9%). The majority of patients presented with pN2-pN3 disease (36 patients, 80.0%). ECS was present in 21 patients (36.2%), and 37 patients (63.8%) had high-grade disease. The majority of affected lymph nodes were located in level II (*n* = 35) or level III (*n* = 10), and the most prevalent histology was squamous cell carcinoma (*n* = 45; 77.6%). While 2 patients (3.4%) exhibited EBV-positive lymph nodes, 7 tumors (12.1%) were tested positive for HPV. Patient characteristics are summarized in Table 1.

**Table 1:** Patient characteristics regarding CCUP patients treated by radiotherapy in our institution between 2008 and 2019 (*n* = 58). If both a clinical nodal status (cN) and a pathological nodal (pN) status were available, only pN was stated

Variable		
Age (median, range)	62 (37–92)	
**Sex**	**n**	**%**
female	14	24.1
male	44	75.9
**ECOG**		
0	7	12.1
1	29	50.0
unknown	22	37.9
**Smoking**		
no	17	29.3
yes	29	50.0
unknown	12	20.7
**Localization**		
I	2	3.4
II	38	65.5
III	10	17.2
IV	2	3.4
VIII	4	6.9
unknown	2	3.4
**cN,*n*** **= 13**		
cN1	1	7.7
cN2	8	61.5
cN3	3	23.1
unknown	1	7.7
**pN, n = 45**		
pN1	9	20.0
pN2	26	57.8
pN3	10	22.2
**cM**		
0	36	62.1
1	1	1.7
x	19	32.8
unknown	2	3.4
**Histology**		
squamous cell carcinoma	45	77.6
adenocarcinoma	2	3.4
undifferentiated	5	8.6
others^1^	4	6.9
unknown	2	3.4
**Grading**		
1	0	0.0
2	14	24.1
3	37	63.8
unknown	7	12.1
**HPV/EBV**		
HPV-positive	7	12.1
EBV-positive	2	3.4
**ECS**		
no	27	46.6
yes	21	36.2
unknown	10	17.2

^1^sarcomatoid carcinoma, lymphoepithelial carcinoma

Nearly all patients received an ultrasound examination (Table 2). CT of the neck and thorax was performed in 45 patients (77.6%), whereas FDG-PET-CT was performed in 30 patients (51.7%). Fifty-two patients were investigated by panendoscopy (89.7%). Treatment included unilateral ND in 23 patients (39.7%) and bilateral ND in 15 patients (25.9%). ND was omitted in 22.4% of patients. Simultaneously or sequentially to panendoscopy/ND, a unilateral or bilateral tonsillectomy was performed in 37 patients (63.8%).

**Table 2:** Diagnostic work-up for CCUP patients treated in our institution between 2008 and 2019

Diagnostics	n	%
**Sonography neck**		
no	2	3.4
yes	53	91.4
unknown	3	5.2
**CT**		
no	6	10.3
head	3	5.2
head/thorax	45	77.6
head/thorax/abdomen	4	6.9
**Panendoscopy**		
no	6	10.3
yes	52	89.7
**GI endoscopy**		
no	15	25.9
yes	42	72.4
unknown	1	1.7
**MRI neck**		
no	32	55.2
yes	25	43.1
unknown	1	1.7
**PET-CT**		
no	28	48.3
yes	30	51.7
**Neck dissection**		
no	13	22.4
neck dissection unilateral	23	39.7
neck dissection bilateral	15	25.9
others^1^	7	12.1
**Tonsillectomy**		
vno	17	29.3
yes	37	63.8
unknown	4	6.9

^1^sampling, lymph node extirpation

### Radiotherapy and chemotherapy characteristics

The majority of patients received adjuvant radiotherapy after lymph node dissection (*n* = 35; 60.3%); 21 patients (36.2%) were treated with definitive radiotherapy, and 2 patients (3.4%) were scheduled for palliative radiotherapy, performed in the setting of oligometastatic disease (Table 3). Radiotherapy was completed in 52 patients (89.7%), with the primary reason for therapy discontinuation being deterioration of the general condition and/or progressive disease. A sequential or simultaneous integrated boost to the macroscopic tumor or tumor bed was applied in 48 patients (82.8%).

**Table 3:** Treatment characteristics in our CCUP patient cohort

Treatment	n/median	%
**Radiotherapy**		
definitive	21	36.2
adjuvant	35	60.3
palliative	2	3.4
**Completion radiotherapy**		
no	4	6.9
yes	52	89.7
unknown	2	3.4
**Radiotherapy dose (median, range)**		
Total dose (including boost)	60.0 Gy (18.0–72.0)	
**Single radiation dose**		
1.7 Gy	1	1.7
1.8 Gy	3	5.2
2 Gy	52	89.7
3 Gy	2	3.4
**Boost,*n*** **= 48**		
integrated	4	8.3
sequential	44	91.7
**Simultaneous chemotherapy**		
no	18	31.0
yes	40	69.0
**Chemotherapy,*n*** **= 40**		
cisplatin	27	67.5
cisplatin/5-fluorouracil	4	10.0
cetuximab	1	2.5
carboplatin	5	12.5
others^1^	3	5.0
**Completion chemotherapy, n = 40**		
no	6	15.0
yes	26	65.0
unknown	8	20.0

^1^mitomycin C/5-fluorouracil, cisplatin/vinorelbine, cisplatin/mitomycin C/5-fluorouracil

Concurrent chemotherapy was prescribed in 40 patients (69.0%); indications for simultaneous chemotherapy were ECS and residual tumor. In 6 patients, concurrent chemotherapy was recommended by the multidisciplinary tumor board for adenocarcinoma or undifferentiated histologies and rapid tumor growth. Twenty-seven patients (67.5%) received cisplatin monotherapy (100 mg/m^2^ body surface area every 3 weeks) (**supplementary table** **1****, supplementary table** **2**). Carboplatin monotherapy was administered in 5 patients (12.5%), and lower-dose cisplatin combined with 5-fluorouracil in 4 patients (10.0%). Cetuximab was applied in 1 case on an individual basis. Twenty-six patients (65.0%) completed the planned chemotherapy cycles. Induction chemotherapy was not performed in any patient.

### Patient outcomes

**Fig. 2:**
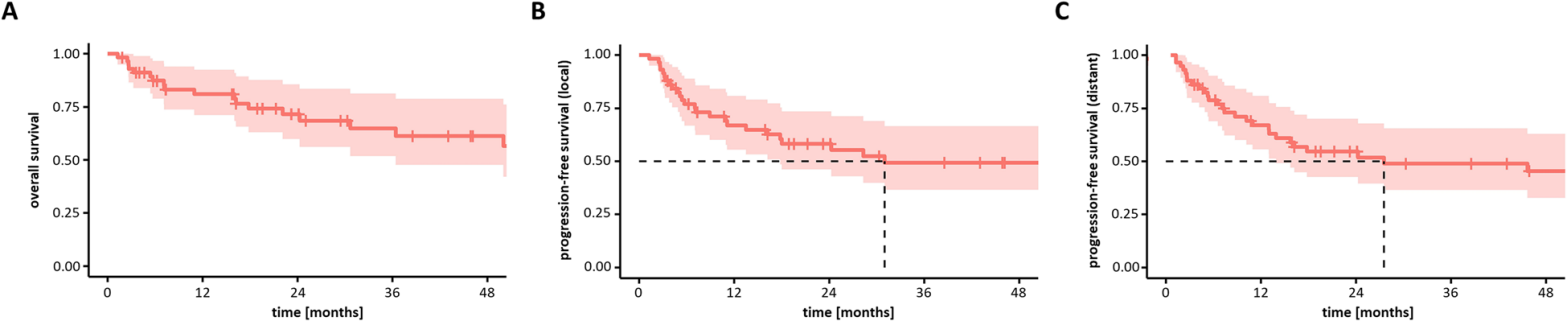
Kaplan-Meier curves showing OS (**a**), PFS (locoregional) (**b**) and DMFS (**c**) of CCUP patients treated by radiotherapy (*n* = 58). The red area shows the 95% confidence intervals for the survival rates

Median follow-up in this patient cohort was 29.9 months (range 4.6–121.9 months). Median OS was not reached, and 1-year OS, 3-year OS and 5-year OS were 81,2, 64.9% and 56,6%, respectively (Fig. 2a). Restricting the analysis to CCUP patients with SCC, 1-year OS, 3-year OS and 5-year OS ranged at 83.3, 68.6 and 62.4%, respectively. There was no significant OS difference between CCUP patients with SCC and non-SCC histology (**supplementary Figure****1**) (*p* = 0.48, log-rank test). Seven patients (12.1%) developed in-field lymph node relapse during the follow-up period, and median locoregional PFS was 31 months, with 1-year PFS, 3-year PFS and 5-year PFS amounting to 70.0, 49.3 and 49.3%, respectively (Fig. 2b). Locoregional PFS exclusively for SCC CCUP was 73.8, 57.9 and 57.9% after 1 year, 3 years and 5 years, respectively. Distant metastases were diagnosed in 13 patients (22.4%) after treatment. Median DMFS was 27.5 months with 1-year DMFS, 3-year DMFS and 5-year DMFS ranging at 67.1, 48.9 and 45.4%, respectively (Fig. 2c). If limited to SCC as CCUP histology, 1-year DMFS, 3-year DMFS and 5-year DMFS amounted to 71.1, 56.7 and 52.6%, respectively.

**Fig. 3:**
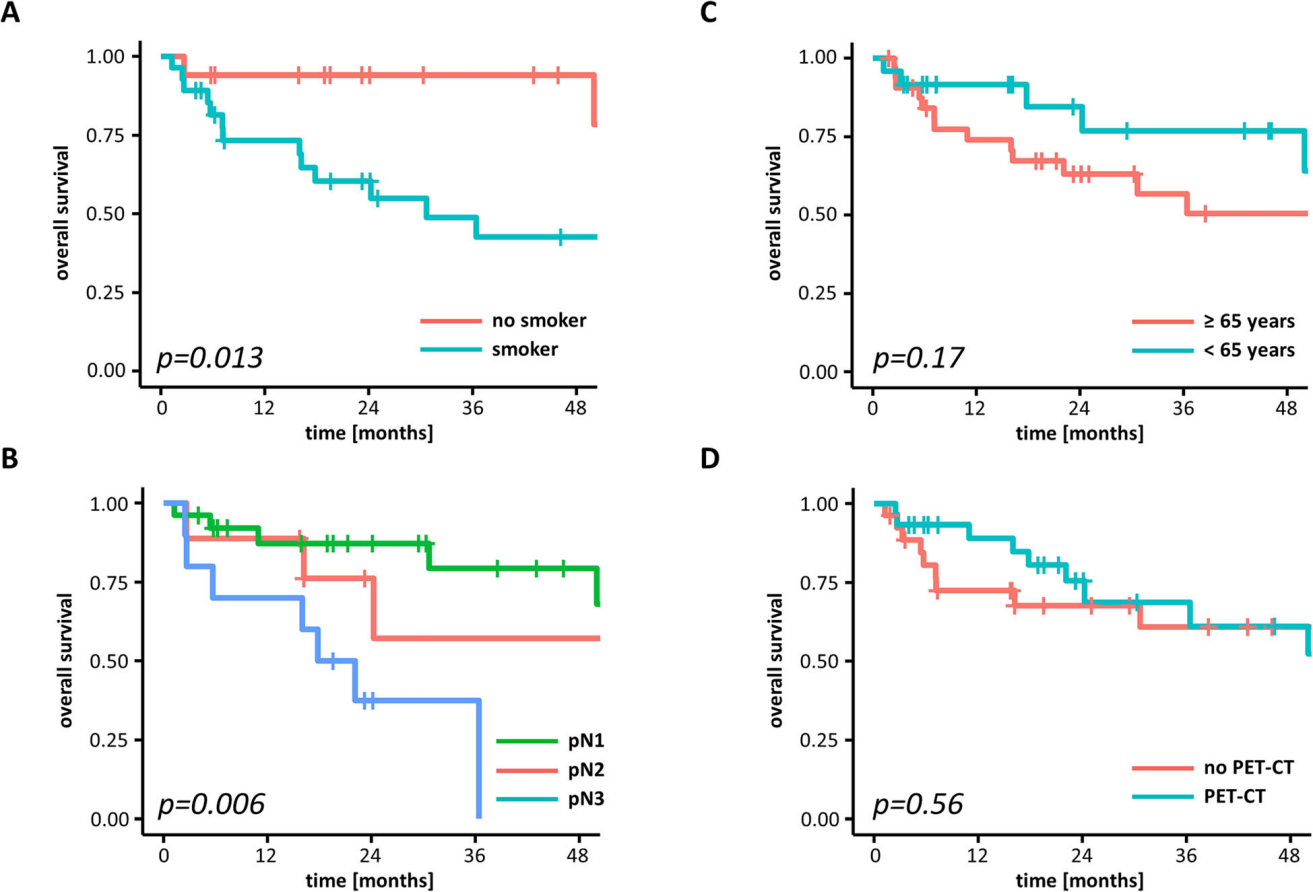
Kaplan-Meier curves demonstrating OS according to smoking status (**a**), pathological nodal status (**b**), age (**c**) and FDG-PET-CT imaging (**d**). Log-rank tests were performed to compare the groups

Univariate analyses demonstrated that smoking was significantly associated with impaired OS (HR = 5.34, 95% CI 1.21–23.55, *p* < 0.05) (Fig. 3a**,** Table 4). There was a trend towards reduced OS for CCUP patients with pN3 (pN3 versus pN1: HR = 3.76, 95% CI 0.91–15.55, *p* = 0.07), and log-rank tests demonstrated significantly reduced OS for advanced nodal status (*p* < 0.05, log-rank tests) (Figs. 3b). Cox regression analyses of age, gender, tumor histology, tumor grading, ECS, utilization of FDP-PET-CT, ND and concomitant chemotherapy did not demonstrate an effect on survival (*p* > 0.05 for all) (Fig. 3c and d).

**Table 4:** Cox-regression for clinical and pathological parameters regarding OS effects

Parameter	HR	CI 95%	***p***-value
Age ≥ 65 / < 65 years	2.04	0.72–5.56	0.18
Sex male / female	1.25	0.36–4.36	0.73
Smoker / non-smoker	5.34	1.21–23.55	0.03
No SCC / SCC	1.72	0.38–7.69	0.49
Grading G3 / G2	1.22	0.34–4.30	0.76
pN3 / pN1	3.76	0.91–15.55	0.07
pN2 / pN1	0.67	0.17–2.70	0.57
pN2b-pN3 / pN1-pN2a	1.49	0.54–4.17	0.43
ECS / no ECS	1.73	0.68–4.39	0.25
No PET-CT / PET-CT	1.30	0.53–3.23	0.56
No ND / unilateral ND	1.52	0.52–4.45	0.45
Bilateral ND / unilateral ND	0.91	0.30–2.78	0.87
No ND / any ND	1.79	0.67–4.76	0.24
No Chemotherapy / chemotherapy	0.63	1.92–2.07	0.42

### Acute toxicities

Acute toxicities were assessed during radiotherapy as well as the first 90 days after completion of treatment and were quantified using National Cancer Institute-Common Terminology Criteria for Adverse Events (CTCAE v4.03). Overall, rates of grade 3 treatment-related acute toxicities were moderate and were observed for radiation dermatitis in 3 patients (5.2%) and for oral mucositis in 12 patients (20.7%) (Table 5). Only 1 patient (1.7%) developed grade 4 mucositis, and there were no grade 5 events. Chronic treatment-related toxicities could not be systematically assessed due to the lack of systematic long-term follow-up information.

**Table 5:** Toxicity results of several (chemo)radiotherapy-related acute side effects according to the Common Terminology Criteria for Adverse Events (CTCAE) v5.0

Toxicity	n	%
**Dermatitis**		
0	9	15.5
1	25	43.1
2	21	36.2
3	3	5.2
**Dysphagia**		
0	8	13.8
1	15	25.9
2	34	58.6
3	1	1.7
**Nausea**		
0	33	56.9
1	22	37.9
2	3	5.2
**Mucositis**		
0	8	13.8
1	9	15.5
2	28	48.3
3	12	20.7
4	1	1.7
**Xerostomia**		
0	9	15.5
1	26	44.8
2	23	39.7
**Hoarseness**		
0	43	74.1
1	15	25.9
**Dyspnea**		
0	54	93.1
1	4	6.9
**Dysgeusia**		
0	9	15.5
1	32	55.2
2	17	29.3
**Pain**		
0	21	36.2
1	25	43.1
2	12	20.7

## Discussion

Our data derived from a large cohort of patients receiving IGRT and mostly IMRT for CCUP demonstrate that radiotherapy as part of a multidisciplinary treatment approach including is an effective treatment modality for these patients and results in relatively high locoregional control rates with moderate higher-grade toxicities. Nevertheless, the data show that systemic control remains a challenge in these patients as reflected in median DMFS rates of only 27.5 months. With a median age of 62 years, predominantly male patients and SCC as the most common histology, our patient cohort is comparable to previous CCUP studies in terms of demographic parameters [2, 18–21]. The majority of lymph node metastases were observed in the upper neck with level II as the most frequent localization, suggesting an occult head-and-neck cancer in the majority of patients [5].

Compared to other retrospective analyses, our oncological results in terms of OS and locoregional control are quite favorable [18]. While older series focused on conventional 2- or 3-dimensional radiotherapy techniques reported 5-year survival rates between 22 and 53%, our cohort exhibited a 5-year OS of almost 60% [4, 22–25]. Our results especially hold up favorably as we did not limit our analysis to squamous cell CCUP, which commonly has a favorable prognosis compared to other cervical histologies [13, 26]. Other studies including only SCC CCUP reported about 5-year OS rates ranging at 40% [19], 47% [27], 48% [21] and 52% [28], which is lower than the 5-year OS of 62% in SCC CCUP patients in our analysis.

Diagnostic tonsillectomy was performed in 63.8% of our CCUP patients. It has been previously demonstrated that tonsillectomy resulted in the diagnosis of primary tonsillar carcinoma in about one quarter of patients that initially presented with CCUP [29]. Additionally, previous data suggest that a diagnostic workup supplementing imaging and panendoscopy with a bilateral tonsillectomy was found to lead to tumor detection rates of 59.6%, which were superior to imaging alone, and survival rates for CCUP were found improved if a tonsillectomy was performed [4, 30].

The relevance of individual diagnostic procedures has remained somewhat controversial, but the widespread utilization of FDG-PET-CT in the last years has increased detection rates of primary cancer sites in CCUP. Several analyses quantifying the use of FDG-PET-CT for CCUP have reported overall primary tumor detection rates ranging between 24.5 and 37% [10, 11]. In our analysis, FDG-PET-CT utilization was not associated with improved OS. On the one hand, this could be due to the lack of power of the hypothesis test, as our sample size is quite small. On the other hand, considering that the majority of CCUP cases are due to underlying head-and-neck cancers, the lack of an effect for FDG-PET-CT imaging could also be due to the extensive coverage of the head-and-neck region including the complete bilateral lymphatics and mucosa in our patient cohort. However, the widespread utilization of PET imaging for CCUP staging may help to de-escalate the treatment volumes to the involved sites or laterality in order to reduce treatment-related toxicities [31]. Furthermore, as utilization of FDG-PET-CT in the diagnostic work-up may have facilitated detection of the primary tumor, some patients with initial suspicion of CCUP may have received de-intensified treatments after detection of the primary tumor. Based on the results from the prospective DAHANCA-13 trial, PET-CT imaging is now recommended as part of the diagnostic workup for CCUP patients [6].

Another controversial topic pertains to the extent of radiotherapy, especially the need for uni- versus bilateral treatment to the elective neck. A meta-analysis failed to demonstrate a survival benefit for elective bilateral neck radiotherapy in comparison to unilateral treatment [32]. However, in a large retrospective Danish study, the 5-year control rates were reported to be 51% for bilateral radiotherapy versus only 27% for ipsilateral radiotherapy accompanied by a trend towards improved disease-specific survival; however, the staging means were not comparable to the current standards [2]. The EORTC-24001-22005 trial aimed to clarify this issue by conducting a prospective randomized trial comparing ipsilateral versus bilateral neck radiotherapy for CCUP patients but was unable to provide results owing to low patient enrollment. A recent meta-analysis reported an improvement regarding locoregional tumor control rates, but not disease-free survival for bilateral radiotherapy compared to ipsilateral radiotherapy [33]. The majority of the published studies did not provide routine FDG-PET-CT-based staging of the neck, and considering the lower sensitivity of other diagnostic imaging modalities, contralateral lymph node metastases may have been missed in a subgroup of patients. Bilateral radiotherapy, especially when combined with radiotherapy to large mucosal areas was reported to provoke high toxicity rates for CCUP patients, but these patients were treated with older 2- or 3-dimensional techniques [33]. Our study focused on patients that were treated with IGRT and IMRT, as these newer techniques have demonstrated superior toxicity profiles for head-and-neck treatments in various analyses [2, 22, 23, 34–36]. Hence, in our cohort, only 5.2% of patients exhibited acute grade 3 radiation dermatitis and 22.4% of patients had acute grade ≥ 3 mucositis with no reported grade 5 toxicities, demonstrating an acceptable tolerance for extended radiotherapy. The moderate acute toxicity rates were mirrored in the high radiotherapy completion rates in about 90% of patients. It should be noted that the mucosal doses varied between 50 and 54 Gy in our cohort, which is lower than in most other series [21, 37, 38]. The current NCCN guideline recommends irradiation doses to the putative mucosal sides ranging at 50–60 Gy in combination with concomitant systemic treatment; if radiotherapy is administered alone, 50–66 Gy are proposed. Therefore, the lower mucosal radiation dose may contribute to the favorable toxicity profile in our study cohort.

In our cohort, positive smoking history was found to significantly impair survival of CCUP patients treated by radiotherapy. Tribius and colleagues reported about an association between smoking and HPV-status in CCUP patients with significantly more HPV-positive tumors in smokers [37]. Furthermore, tobacco history negatively affected survival of patients with HPV-positive CCUPs in this study. As HPV-testing was not routinely performed in the time span of our study, we were not able to investigate the relationship between smoking history and HPV-status in CCUP patients. However, smoking should be considered as a risk factor for CCUP patients, especially in the case of HPV-positive tumors. In a study performed by Dixon and coworkers, CCUP patients with p16-positive tumors exhibited less advanced nodal status and superior disease-free survival [39]. The observed trend towards improved survival in CCUP patients with N1-status may therefore also partially related to the HPV-status.

Whether radiotherapy can be omitted altogether after primary surgery is another matter of debate. Especially for pN1 or pN2a stages without ECE, omission of radiotherapy may be justified if a close and imaging-based follow-up is guaranteed, so that salvage radiotherapy can be applied in case of progression. However, in a large study comprising data from 5 Danish centers, patients treated with surgery alone exhibited a significantly elevated risk of emerging primary compared to those receiving adjuvant radiotherapy [2]. 5-year risk for emerging primary was 54% in the surgery group versus 15% in the adjuvant RT group [2]. Therefore, to date, postoperative radiotherapy is warranted for the large majority of CCUP patients.

The use and benefit of concomitant chemoradiotherapy in CCUP patients has been a matter of debate and largely lacks clinical evidence due to missing prospective studies [5, 40]. The indications for the utilization of concomitant systemic treatment are extrapolated from data on other head-and-neck cancers, and platinum-based agents are most commonly used for concomitant chemotherapy [40, 41]. In a study by Chen and colleagues, concomitant chemotherapy was found to result in increased toxicity rates but no improvements of OS, PFS or locoregional control rates [40]. Due to the retrospective nature of this study, it should be noted that simultaneous chemotherapy may have been applied especially for CCUPs with high-risk features or more advanced disease, suggesting a potential bias to the disadvantage of concurrent chemotherapy utilization. In our cohort, chemotherapy was most commonly prescribed for macroscopic tumor, ECS or positive resection margins according to the established high-risk features for head-and-neck SCC, but did not result in an improvement of patient survival [42–44].

While our analysis is one of the first comprehensive datasets presenting outcome and toxicities in CCUP patients undergoing IGRT and mainly IMRT, it has several limitations due to its retrospective nature and small sample size. Prospective studies for relatively rare disease constellations are difficult to conduct, and especially prospective studies comparing radiotherapy with surgery exhibit a high risk of failure due to insufficient accrual [45]. However, despite the aforementioned challenges, prospective, multi-center trials will be eventually needed in order to identify the ideal diagnostic and treatment algorithm for CCUP patients. Additionally, regarding the occurrence of distant metastases in almost 1 of 4 CCUP patients, further research will need to focus on effective systemic treatments as part of adjuvant therapy in order to avoid distant relapse.

Taken together, out dataset demonstrated excellent results and moderate toxicity in CCUP patients when treated with extensive radiotherapy based on modern radiation techniques.

## Supplementary information

**Additional file 1 Supplementary Figure 1.** Kaplan-Meier curves showing OS for non-SCC CCUP and SCC CCUP patients. The *p*-value indicates the result of the log-rank test.

**Additional file 2.** Supplementary Table 1: Administration of concomitant chemotherapy depending on the histology of CCUP.

**Additional file 3.** Supplementary Table 2: Specification of concomitant chemotherapy in dependence of the CCUP histology.

## Data Availability

The datasets used and analyzed during the current study are available from the corresponding author on reasonable request.
