# Supplementary figures and images for: Radiotherapeutic management of cervical lymph node metastases from an unknown primary site – experiences from a large cohort treated with modern radiation techniques
Source: Radiat Oncol. 2020 Apr 15;15:80. doi: 10.1186/s13014-020-01529-z (PMC7158130; doi:10.1186/s13014-020-01529-z)

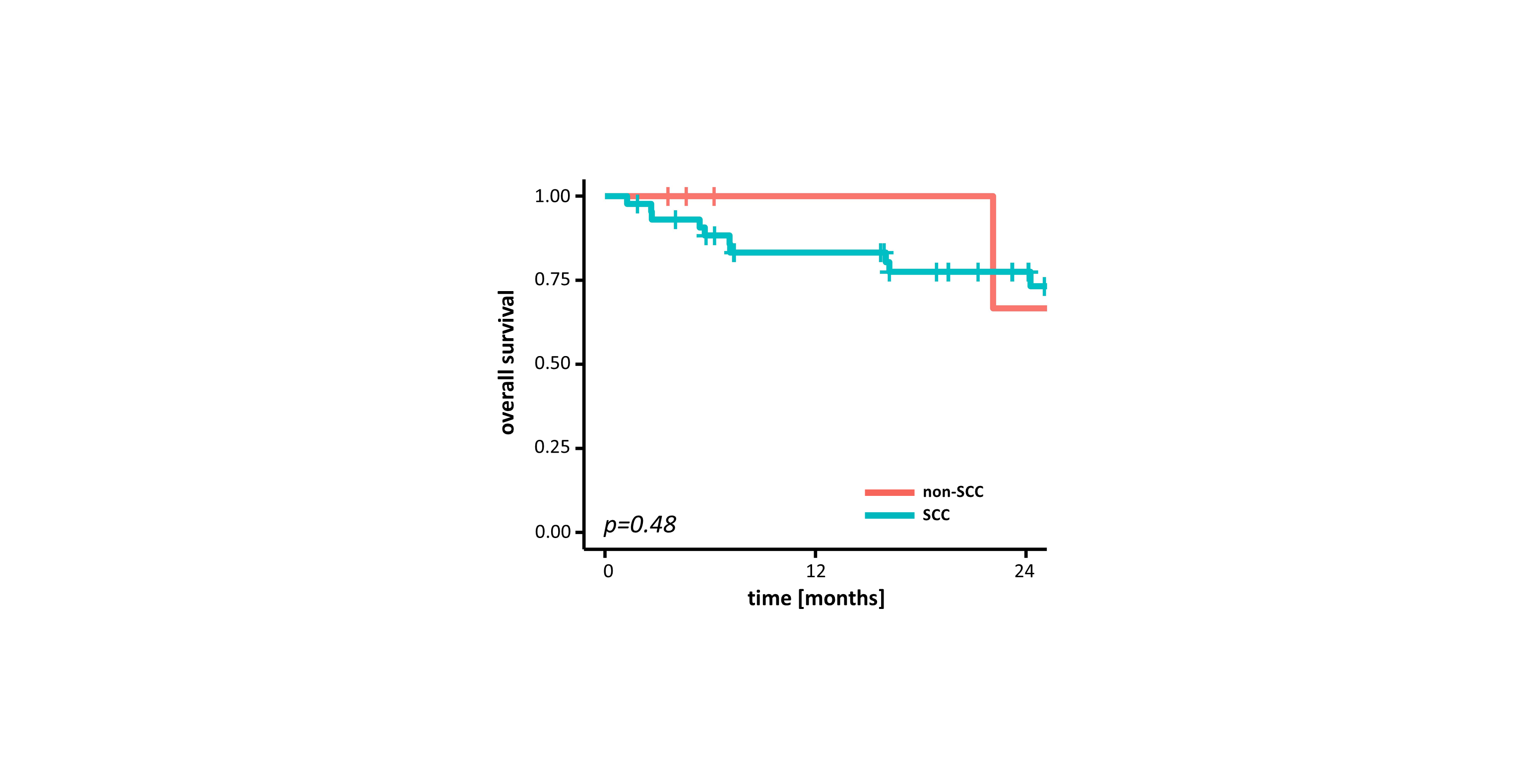

Supplement: Supplementary file 1 — Additional file 1 Supplementary Figure 1. Kaplan-Meier curves showing OS for non-SCC CCUP and SCC CCUP patients. The p-value indicates the result of the log-rank test. [file 13014_2020_1529_MOESM1_ESM.tif]
