# Supplementary material for: Radiotherapeutic management of cervical lymph node metastases from an unknown primary site – experiences from a large cohort treated with modern radiation techniques
Source: Radiat Oncol. 2020 Apr 15;15:80. doi: 10.1186/s13014-020-01529-z (PMC7158130; doi:10.1186/s13014-020-01529-z)
Supplement: Supplementary file 2 — Additional file 2. Supplementary Table 1: Administration of concomitant chemotherapy depending on the histology of CCUP. [file 13014_2020_1529_MOESM2_ESM.docx]

Supplementary figure 1: Administration of concomitant chemotherapy depending on the histology of CCUP.

|  | **Concomitant chemotherapy** | **No concomitant Chemotherapy** |
| --- | --- | --- |
| **squamous cell carcinoma** | 31 | 14 |
| **adenocarcinoma** | 2 | 1 |
| **undifferentiated** | 2 | 2 |
| **others** | 3 | 1 |
| **unknown** | 2 | 0 |
