# Supplementary material for: Radiotherapeutic management of cervical lymph node metastases from an unknown primary site – experiences from a large cohort treated with modern radiation techniques
Source: Radiat Oncol. 2020 Apr 15;15:80. doi: 10.1186/s13014-020-01529-z (PMC7158130; doi:10.1186/s13014-020-01529-z)
Supplement: Supplementary file 3 — Additional file 3. Supplementary Table 2: Specification of concomitant chemotherapy in dependence of the CCUP histology. [file 13014_2020_1529_MOESM3_ESM.docx]

Supplementary figure 2: Specification of concomitant chemotherapy in dependence of the CCUP histology.

|  | **Cisplatin** | **Cisplatin/5-FU** | **Cetuximab** | **Carboplatin** | **Others** |
| --- | --- | --- | --- | --- | --- |
| **squamous cell carcinoma** | 23 | 1 | 1 | 4 | 2 |
| **adenocarcinoma** | 0 | 0 | 0 | 1 | 1 |
| **undifferentiated** | 1 | 1 | 0 | 0 | 0 |
| **others** | 1 | 2 | 0 | 0 | 0 |
| **unknown** | 2 | 0 | 0 | 0 | 0 |
